# Molecular regulation of functions of *Pseudomonas protegens* by primary metabolites in the rhizosphere: Systematic analyses and applications to agriculture

**DOI:** 10.5511/plantbiotechnology.25.0424a

**Published:** 2025-09-25

**Authors:** Kasumi Takeuchi, Shigemi Seo

**Affiliations:** 1 Institute of Agrobiological Sciences, National Agriculture and Food Research Organization, 2-1-2 Kannondai, Tsukuba, Ibaraki 305-8518, Japan

**Keywords:** disease control, primary metabolites, pseudomonads, rhizosphere, secondary metabolites

## Abstract

*Pseudomonas protegens* and related strains exert protective effects in plants by producing a wide range of secondary metabolites and extracellular enzymes that contribute to the suppression of plant pathogens in the rhizosphere. Our genomic and metabolomic studies on *P. protegens* demonstrated that intracellular low-molecular-weight effectors, such as guanosine tetraphosphate, fumarate, and γ-aminobutyrate, function as important signals in niche adaptation to plant roots. Extra- and intracellular levels of fumarate and succinate correlated with the regulation of secondary metabolism. We then investigated the involvement of exogenous amino acids in plant protection by *P. protegens* against *Pythium* damping off and root rot in cucumber. Among the amino acids tested, glutamate exerted positive effects on the efficacy of plant protection by *P. protegens*. The promoter activities of genes involved in the regulation of functions were characterized in detail, and we noted the dose-dependent regulation of functions in response to exogenous glutamate. In this mini-review, we introduce our previous findings on pseudomonads in terms of effective and ecological applications of this bacterium. The effective regulation of root-colonizing pseudomonads in the rhizosphere using extracellular signals that affect biocontrol activity will lead to advances in research on plant-microbe interactions.

## Introduction

Plant diseases are of global concern because of risks to food security, human health and environmental sustainability. According to FAO, annually up to 40 percent of global crop production is lost due to plant pests and diseases (https://www.fao.org/plant-production-protection/about/en (Accessed Apr 14, 2025)). Therefore, sustainable plant disease management is critical to meet food security needs while reducing environmental impact. Soil-borne diseases, such as damping off and root rot, caused by *Pythium* species, which are pathogenic oomycetes, significantly reduce the yields of the agricultural systems of many crops and are difficult to control, especially because *Pythium* species can develop resistance to certain fungicides (Arora et al. 2021; Janvier et al. 2007; Wille et al. 2019). *Pythium* species have a broad host range, affecting many plant families and it is estimated that *Pythium* diseases are responsible for billion-dollar losses per year around the world (van West et al. 2003).

These issues have led to recent advances in research on biocontrol agents that utilize beneficial microorganisms represented by plant growth-promoting rhizobacteria (PGPR). Many PGPR isolates in the genera *Bacillus* and *Pseudomonas* have been employed as biocontrol agents (Dimkić et al. 2022; Niu et al. 2020). Root-colonizing fluorescent pseudomonads classified into the *Pseudomonas fluorescens* group, represented by *P. protegens*, produce a number of antibiotic secondary metabolites and extracellular enzymes that contribute to the suppression of pathogenic fungi, nematodes, and insects; therefore, they are regarded as effective biocontrol strains against plant diseases (Howell and Stipanovic 1979; Ramette et al. 2011; Stutz et al. 1986). *P. protegens* has been particularly reported to be effective for the control of soil-borne diseases in both monocots and dicots (Keel et al. 1992; Subramoni et al. 2011). In this mini-review, we introduce our metabolomic research findings on *P. protegens* and our perspectives to improve the efficacy of plant protection against soil-borne diseases by utilizing primary metabolites in the rhizosphere.

## Metabolomic profiling of strain CHA0 and Gac/Rsm mutants

**Figure 1:**
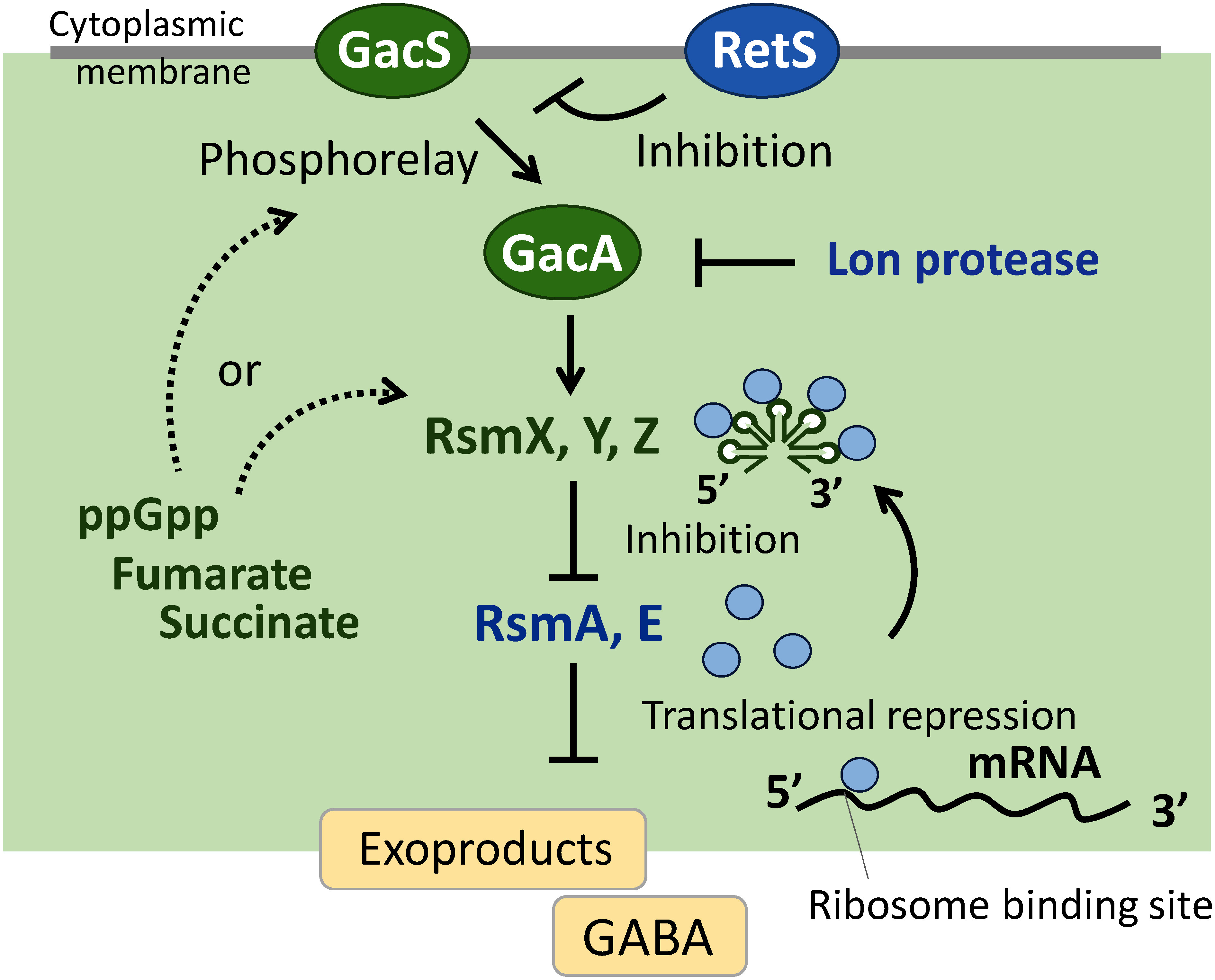
Figure 1. A schematic model for gene regulation by primary metabolites in the Gac/Rsm system of *Pseudomonas protegens* CHA0. Evidence for this model is based on previous studies (Heeb et al. 2002, 2005; Kay et al. 2005; Reimmann et al. 2005; Takeuchi 2018; Takeuchi et al. 2009, 2012, 2014b; Valverde et al. 2003).

The *P. protegens* strain CHA0 (previously called *P. fluorescens* CHA0) has been used as a model strain in studies on the biosynthesis of secondary metabolites, such as 2,4-diacetylphloroglucinol and pyoluteorin, which exhibit antibiotic activities in the rhizosphere (Haas and Keel 2003). These exoproducts contribute to plant protection by these strains and other root-colonizing *Pseudomonas* species with biocontrol activity. The expression of these biocontrol factors depends on the Gac/Rsm signal transduction pathway, which is elicited by the GacS/GacA two-component system (Lapouge et al. 2008; Valentini and Filloux 2016). Activated GacA then promotes the transcription of non-coding small RNAs (sRNAs). These sRNAs have high affinity for the RNA-binding proteins RsmA and RsmE. RsmA/E proteins repress the translation of genes involved in secondary metabolism during trophophase. Therefore, when sRNAs are induced, they relieve the translational repression of target genes by sequestering the RsmA/E proteins, thereby allowing the synthesis of secondary metabolites. A schematic model for this system is summarized in Figure 1. Since sRNA expression levels correlate with the production level of antibiotics, sRNAs are regarded as effective biomarkers for elucidating the function of secondary metabolism in *P. protegens*. In this context, we examined the expression of the sRNA RsmZ by utilizing *rsmZ-gfp* and *rsmZ-lacZ* reporter constructs and identified genetic and metabolic factors affecting the expression of these constructs.

To identify metabolites involved in the function of the Gac/Rsm system, we examined the intracellular metabolome in wild-type CHA0 and the *gacA* and *retS* mutants using CE-TOFMS (Takeuchi et al. 2012). In *P. protegens* and *P. aeruginosa*, RetS inhibits the activity of the Gac/Rsm pathway by forming a heterodimeric complex with GacS, thereby affecting the expression of target genes and biocontrol factors (Goodman et al. 2009; Humair et al. 2009). Through the metabolome study mentioned above, we investigated the extent to which the metabolomic profile of strain CHA0 was affected in a *gacA-*negative mutant, which lost Gac/Rsm activities, and in a *retS*-negative mutant, which exhibited markedly enhanced Gac/Rsm-dependent activities. Among the metabolites regulated by the Gac/Rsm system, we focused on the intracellular alarmone guanosine tetraphosphate (ppGpp) and GABA, a four carbon non-proteinogenic amino acid, because these two metabolites were the most strongly regulated by GacA. Moreover, ppGpp has been identified as a regulator of virulence in various bacteria (Braeken et al., 2006; Dalebroux et al. 2010; Potrykus and Cashel 2008). We conducted a detailed characterization by constructing a *relA spoT* double mutant, which is deficient in ppGpp synthesis. A reduction in the expression of Rsm sRNAs was noted in this mutant, and antibiotic production, root colonization, and plant protection were all markedly diminished. Therefore, ppGpp appeared to be essential for sustaining the epiphytic fitness and biocontrol activity of strain CHA0 (Takeuchi et al. 2012). Since GABA accumulated in the *gacA* mutant and was depleted in the *retS* mutant, we focused on the *gabT* gene, which encodes for a GABA transaminase that generates succinic semialdehyde (Takeuchi 2018). In that study, we examined the regulatory mechanisms of GABA accumulation in *P. protegens* CHA0 by constructing the *gabT* mutant, which showed a higher level of GABA accumulation. The putative Shine–Dalgarno sequence of *gabD*, which is located upstream of *gabT* and predicted to be co-transcribed and co-translated with *gabT*, has a potential RsmA/E binding site, which may expose the conserved **A**A**GGA**A hexa-loop and allow for the binding of RsmA/E (Lapouge et al. 2008). In support of this finding, promoter activity was significantly weaker in the *gacA* mutant than in the wild type. The importance of this finding is that a post-transcriptional mechanism that enabled the Gac/Rsm system to regulate secondary metabolism in previous studies was shown to regulate a reaction in primary metabolism. GABA also promoted a planktonic lifestyle and reduced biofilm formation by *P. protegens* CHA0. In this context, GABA chemotaxis was required for the virulence of the phytopathogenic bacterium *Pseudomonas syringae* pv. *tabaci* 6605 (Tumewu et al. 2020), demonstrating that this molecule is crucial for interactions between plants and plant-associated bacteria as well as its potential involvement in root colonization by *P. protegens* CHA0 (Takeuchi 2018).

## Exogenous treatment with amino acids and the function of *P. protegens* in the *Pythium*-cucumber pathosystem

The findings from the intracellular metabolome analysis prompted us to search for extracellular signal(s) that effectively regulate the function of *P. protegens*. In consideration of effective applications for biocontrol research, the identification of extracellular signals that affect plant protection efficacy is important. In a random mutagenesis study, we showed that the extra- and intracellular levels of intermediates of the Krebs cycle, including fumarate, positively regulated secondary metabolism through sRNA expression in *P. protegens* (Takeuchi et al. 2009). In that study, fumarate and succinate showed positive effects on the sRNA RsmZ expression, assessed by *rsmZ-gfp* reporter construct, suggesting that fumarate and succinate exert a critical trigger function in secondary metabolism. From a biocontrol point of view, it is interesting to note that these organic acids are major components of root exudates (Kamilova et al. 2006; Sharma et al. 2020). However, the *fumA* mutant, which lacks fumarase and, thus, accumulates fumarate, did not exhibit higher plant protection efficacy than the wild type, but exhibited a higher level of antibiotic activity (Takeuchi et al. 2009). These findings suggest the presence of other signals that effectively induce plant protection; therefore, we searched for other primary metabolites. We summarized the effects of metabolites on the phenotypes of *P. protegens* in the Table 1 and Figure 1.

**Table 1:** Table 1. Effects of metabolites on phenotypes of *Pseudomonas protegens*.

Metabolites	Function
Fumarate	sRNA expression and antibiotics production (+)
Succinate	sRNA expression (+)
Pyruvate	sRNA expression (−)
GABA	Root colonization (+)
ppGpp	sRNA expression, antibiotics production, and plant protection efficacy (+)
Glutamate	Chitinase expression and plant protection efficacy (+)

**Figure 2:**
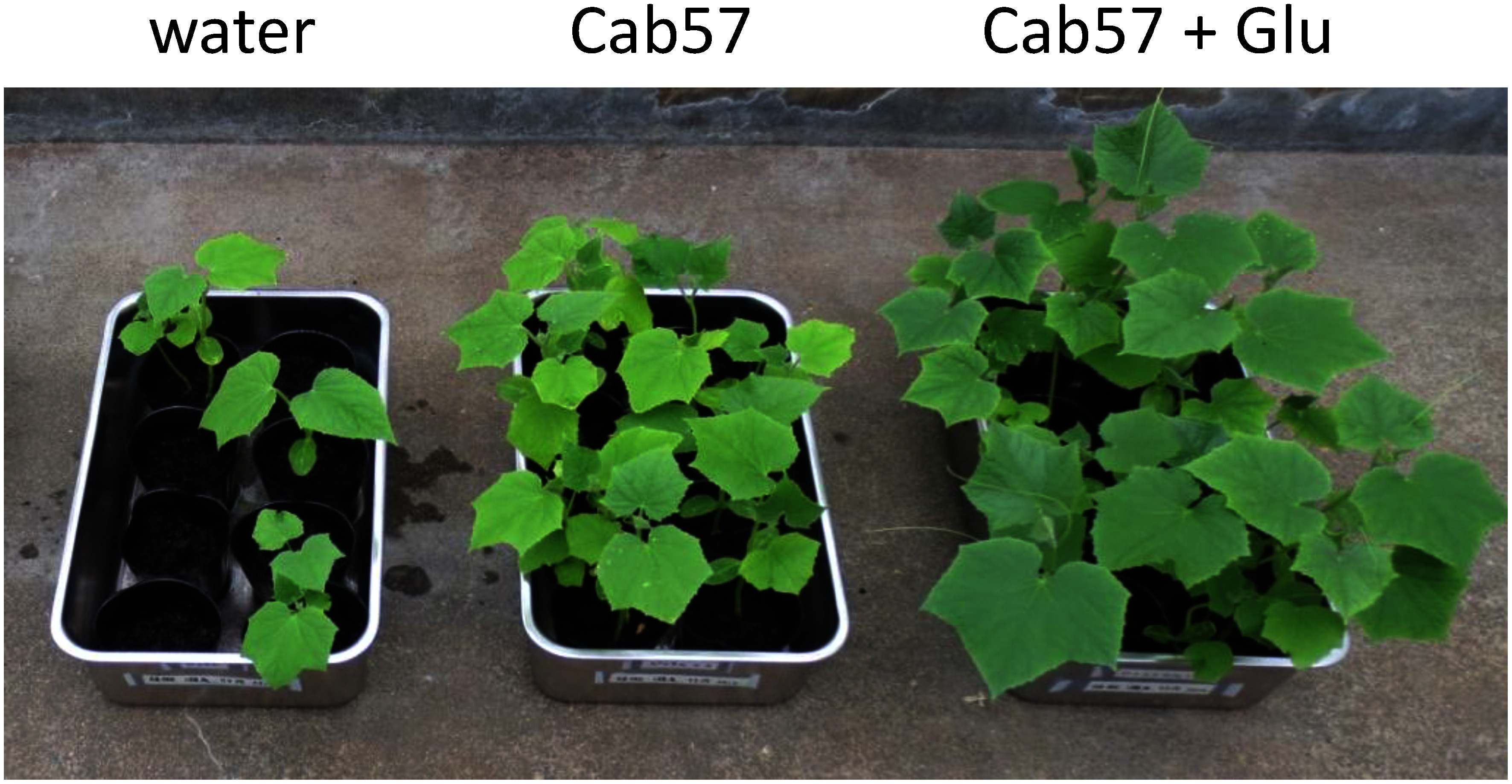
Figure 2. Effects of glutamate on the inhibition of *Pythium* damping off and root rot in cucumber by *Pseudomonas protegens* Cab57. A photo of cucumber plants taken 30 days after planting in *Pythium*-infested soil. (Left) Naturally infested soil, (middle) strain Cab57-treated soil, and (right) strain Cab57- and glutamate-treated soil.

Amino acids have recently been reported to induce plant resistance to pathogens (Goto et al. 2020; Kadotani et al. 2016; Seo et al. 2016). Histidine was shown to function as a plant activator against the bacterial tomato pathogen *Ralstonia solanacearum* when applied to plant roots at a low millimolar level. To investigate the exogenous effects of amino acids on the function of *P. protegens* in a natural habitat, we adopted the cucumber-*Pythium ultimum* pathosystem, which enabled us to evaluate the efficacy of plant protection by measuring root and shoot weights. We examined the efficacy of our domestic strain Cab57 (Takeuchi et al. 2014a) using plastic pots, applied each amino acid to soil to a final concentration of *ca.* 5 mM in the rhizosphere, and incubated the pots in a growth chamber for two weeks (Takeuchi et al. 2023). In that study, we tested 11 amino acids in the L-configuration and only glutamate markedly enhanced the efficacy of strain Cab57. When *Pythium* was infested, the addition of glutamate itself had no effect on the suppression of disease, suggesting that glutamate positively regulated the function of *P. protegens*. We then investigated whether glutamate affected the efficacy of strain Cab57 in a greenhouse for a longer period (one month). When strain Cab57 was added together with glutamate, the efficacy of plant protection was still higher than that with strain Cab57 itself (Figure 2). An RNA-Seq analysis of strain CHA0 revealed that glutamate exerted positive effects on chitinase activity, which may contribute to the efficacy of plant protection (Takeuchi et al. 2023).

Glutamate is present in natural soil at a low micromolar level, but is highly abundant among proteinogenic amino acids (Moe 2013). Furthermore, the concentrations of endogenous glutamate in plant leaves were previously reported to reach approximately 50 mM at damaged sites (Toyota et al. 2018). Assuming that similar conditions occur in natural roots, glutamate released from damaged roots may trigger the function of chitinase in plant protective pseudomonads. Previous findings indicated that glutamate functioned as a signal to induce chitinase activity in rice plants (Kadotani et al. 2016). It has also been reported to prime chitin-induced responses in *Arabidopsis* (Goto et al. 2020). Taken together with our results on *P. protegens*, these findings indicate the relevance of glutamate and chitin-related responses not only in plants, but also in root-colonizing pseudomonads. Therefore, glutamate may function as a signal molecule for disease suppression by both sides of plants and pseudomonads.

**Figure 3:**
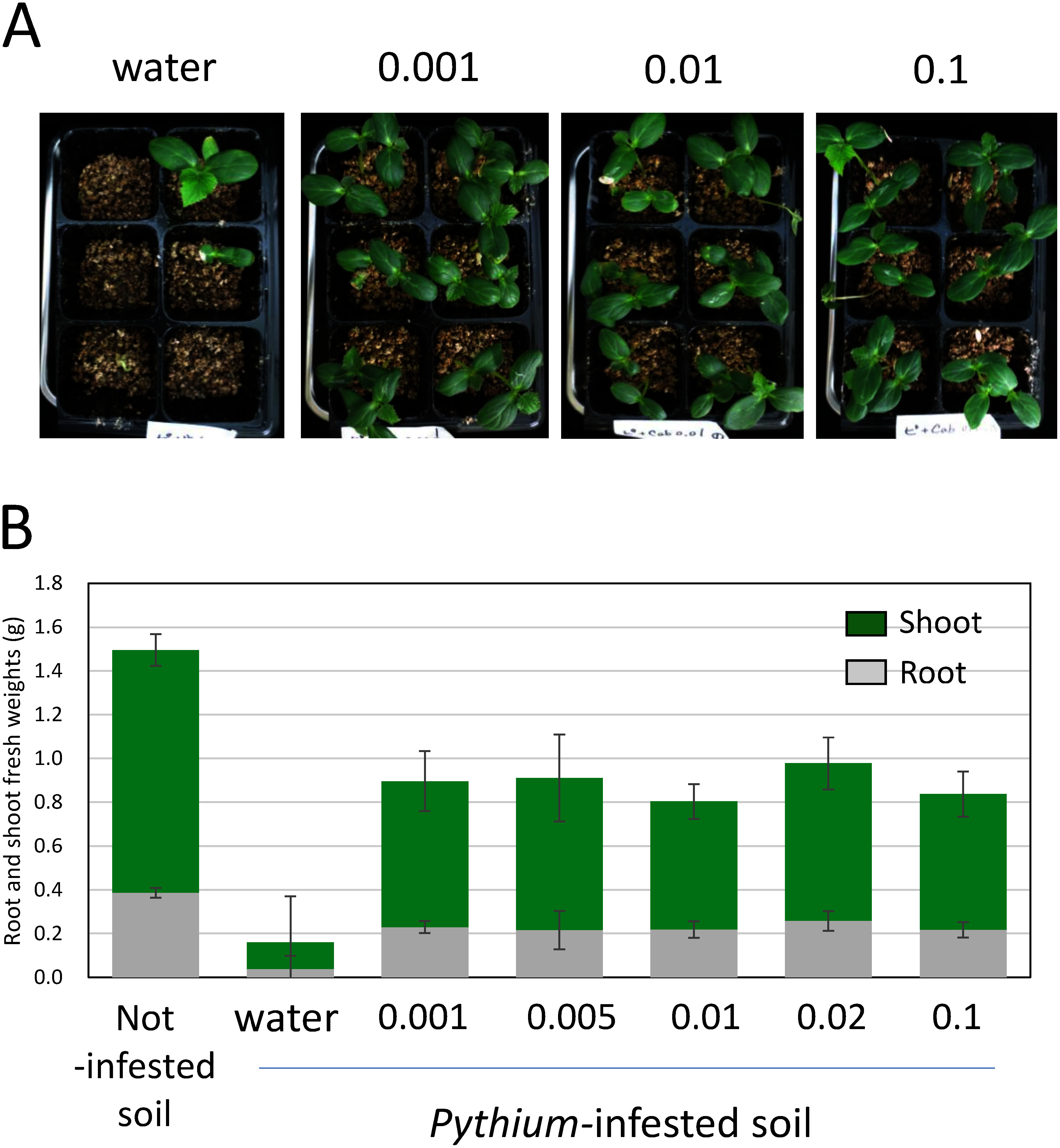
Figure 3. The suppression of *Pythium* damping off and root rot in cucumber by *Pseudomonas protegens* Cab57 at a lower inoculum intensity. (A) A photo of cucumber plants planted in *Pythium*-infested soil. (B) Root and shoot fresh weights in each pot were measured. Data represent the means of two individual repetitions of the same experimental set-up, with 6 replicates (pots containing three cucumber plants) per treatment in each experiment.

In consideration of potential applications, we examined the inoculum intensity of *P. protegens* Cab57 in more detail. The inoculum intensity of *P. protegens* is typically set at an OD_600_ of 0.1 and *ca.* 5 ml of the suspension is added to 25–50 g of soil in plastic pot to reach 10^7^ CFU per gram of soil (Takeuchi et al. 2014a, 2023). We investigated the effects of strain Cab57 on *Pythium* damping off and root rot at lower inoculum intensities (OD_600_ of 0.01 and 0.001). As shown in Figure 3, strain Cab57 protected the plant to a similar extent at the normal inoculum intensity (OD_600_ of 0.1) in both cases, suggesting the effective and practical use of this strain for disease control in the rhizosphere.

## Potential applications of amino acids for disease control and future perspectives for biocontrol research

Amino acids and their related compounds are attracting interest for the control of diseases in crops because of their potential to reduce the environmental burden associated with crop protection. Previous studies demonstrated the protective effects of the exogenous application of amino acids or their related compounds on crop diseases. Glutamate and histidine have been shown to induce resistance in many plants, such as *Arabidopsis*, tobacco, tomato, and rice, to fungal and bacterial pathogens (Goto et al. 2020; Kadotani et al. 2016; Seo et al. 2016; Sun et al. 2019; Yariyama et al. 2019). Exogenously applied phenylalanine induced resistance in chrysanthemum flowers to *Botrytis cinerea* through the production of volatile antibiotic compounds (Kumar et al. 2020). Furthermore, the foliar application of specific amino acids, such as cysteine and glutamate, to cabbage leaves limited invasion by *P. cannabina* pv. *alisalensis*, a bacterial pathogen, into the stomata, which suppressed disease development (Sakata et al. 2023). Aminobutyric acid (ABA), a non-proteinogenic amino acid, has three isomers: α-ABA, β-ABA (BABA), and γ-ABA (GABA). BABA and GABA have been shown to induce disease resistance in many plants (Balmer et al. 2019; Tarkowski et al. 2020). Specific amino acids exert positive effects on crop protection against diseases by changing the microbiota surrounding plants. The inhibition of bacterial wilt disease in tomato caused by *R. solanacearum* in soil treated with lysine or serine was previously attributed to a decline in the bacterial population rather than inhibitory effects through the induction of host resistance (Posas and Toyota 2010). Furthermore, the spray application of glutamate to strawberry- or tomato-enriched populations of PGPR, such as *Streptomyces* in the microbiota surrounding these plants, inhibited the development of fungal diseases (Kim et al. 2021).

Amino acids may also have a negative impact on crop protection. The exogenous application of glutamine and aspartate inhibited BABA-induced disease resistance in tomato (Janotík et al. 2022; Wu et al. 2010). Moreover, *R*. *solanacearum* was shown to promote its pathogenicity by utilizing glutamate from host plants (Shen et al. 2020). Therefore, appropriate management is needed when using amino acids and related compounds for crop protection.

We recently demonstrated that glutamate induced chitinase activity and positively affected the expression of other Gac/Rsm-regulated genes in *P. protegens* CHA0 (Takeuchi et al. 2023). These findings provide support for the positive effects of glutamate on the efficacy of plant protection by *P. protegens* Cab57. Pseudomonads strains have been increasingly marketed as biocontrol agents (Kupferschmied et al. 2013). We recently reported that glutamate promoted root colonization by *P. rhodesiae* HAI-0804, a biocontrol agent (Takeuchi et al. 2024). From a practical point of view, the proper utilization of inexpensive amino acids will contribute to the control of root-colonizing pseudomonads through the regulation of gene expression and rhizosphere colonization. Including these carbon sources in the growth media during the process for manufacturing *Pseudomonas*-based biocontrol agents may be of practical interest for boosting the function of bacteria. Adding amino acids to formulation mixtures of biocontrol agents will be effective for sustainable field applications. Root exudate consisting organic compounds preferable for the behaviors of pseudomonads will also contribute to optimal disease control. Considering the timing of planting and the combination of crops and biocontrol agents amended with amino acids will be critical strategies to engineer the rhizosphere.

## Abbreviations

GABA: γ-aminobutyrate
PGPR: plant growth-promoting rhizobacteria
